# Optogenetic Control of Targeted Peripheral Axons in Freely Moving Animals

**DOI:** 10.1371/journal.pone.0072691

**Published:** 2013-08-21

**Authors:** Chris Towne, Kate L. Montgomery, Shrivats M. Iyer, Karl Deisseroth, Scott L. Delp

**Affiliations:** 1 Department of Bioengineering, Stanford University, Stanford, California, United States of America; 2 Department of Psychiatry and Behavioral Sciences, Stanford University, Stanford, California, United States of America; 3 Howard Hughes Medical Institute, Stanford University, Stanford, California, United States of America; 4 Department of Mechanical Engineering, Stanford University, Stanford, California, United States of America; French National Centre for Scientific Research, France

## Abstract

Optogenetic control of the peripheral nervous system (PNS) would enable novel studies of motor control, somatosensory transduction, and pain processing. Such control requires the development of methods to deliver opsins and light to targeted sub-populations of neurons within peripheral nerves. We report here methods to deliver opsins and light to targeted peripheral neurons and robust optogenetic modulation of motor neuron activity in freely moving, non-transgenic mammals. We show that intramuscular injection of adeno-associated virus serotype 6 enables expression of channelrhodopsin (ChR2) in motor neurons innervating the injected muscle. Illumination of nerves containing mixed populations of axons from these targeted neurons and from neurons innervating other muscles produces ChR2-mediated optogenetic activation restricted to the injected muscle. We demonstrate that an implanted optical nerve cuff is well-tolerated, delivers light to the sciatic nerve, and optically stimulates muscle in freely moving rats. These methods can be broadly applied to study PNS disorders and lay the groundwork for future therapeutic application of optogenetics.

## Introduction

Peripheral nerves transfer information between the central nervous system and the environment, mediating processes as diverse as pain perception and muscle activation. The ability to control targeted sub-populations of peripheral axons in freely moving animals would enable novel experiments to investigate the processes mediated by these axons and could have therapeutic potential. Optogenetics uses light-sensitive ion channels and pumps (typically from the microbial opsin gene family) to control neural activity with high temporal and spatial precision [1]. While optogenetics has been used to great effect in the brain [2], its application in the peripheral nervous system (PNS) has been limited to a few studies [3]–[7]. Previous work in our laboratory has described the first use of optogenetics to activate [4] and inhibit [7] motor neuron axons in anesthetized transgenic mice. These studies demonstrated the application of optogenetics in the PNS, but were limited by an inability to deliver opsins to target cell populations and deliver light for control of behavior in awake and freely moving animals.

The selective expression of opsins within neural sub-populations is an important advantage of optogenetic neuromodulation. Transgenic approaches have been used to express opsins in defined neural populations [8]–[10]. Greater specificity may be achieved through either viral or genetic Cre recombinase-based strategies [11], [12] or through targeting axonal projections via retrograde transport [13]. The latter approach is appropriate for transduction of peripheral neurons, as there exist many recombinant vectors that can transduce such neurons via retrograde transport. Adeno-associated virus (AAV), lentivirus, and herpes simplex virus have been injected into peripheral tissues to target subtypes of sensory and motor neurons by retrograde transport from the axon terminals [14]–[17]. We reasoned that we could adopt these methods to deliver opsins to sub-populations of peripheral motor neurons.

Light delivery in awake and freely moving animals has been another essential component of optogenetic examination of brain circuits. Many peripheral nerve functions cannot be adequately studied in anesthetized animals, but delivering light to peripheral nerves in awake animals is challenging due to the movement of nerves during animal locomotion. Previous studies using chronic electric stimulation of peripheral nerves have faced the same problem and have overcome it through the use of biocompatible spiral nerve cuffs that wrap around the nerve [18].

Here we present an approach to target opsins to sub-populations of motor neurons and to activate their axons using an implantable light delivery system. We photosensitize axons of selected motor neuron pools through intramuscular delivery and subsequent retrograde transport of AAV serotype 6 (AAV6) carrying a channelrhodopsin-2 (ChR2) gene. We implant a biocompatible optical fiber-based nerve cuff that wraps around the sciatic nerve to facilitate muscle-specific ChR2 activation in awake and freely moving animals. The approach we describe enables optogenetic control of peripheral motor axons, can be adapted to other peripheral nerves, and represents a novel gene therapy strategy based on optogenetic neuromodulation.

## Results

### ChR2 delivery to targeted motor neuron axons via AAV6 retrograde transport

**Figure 1:**
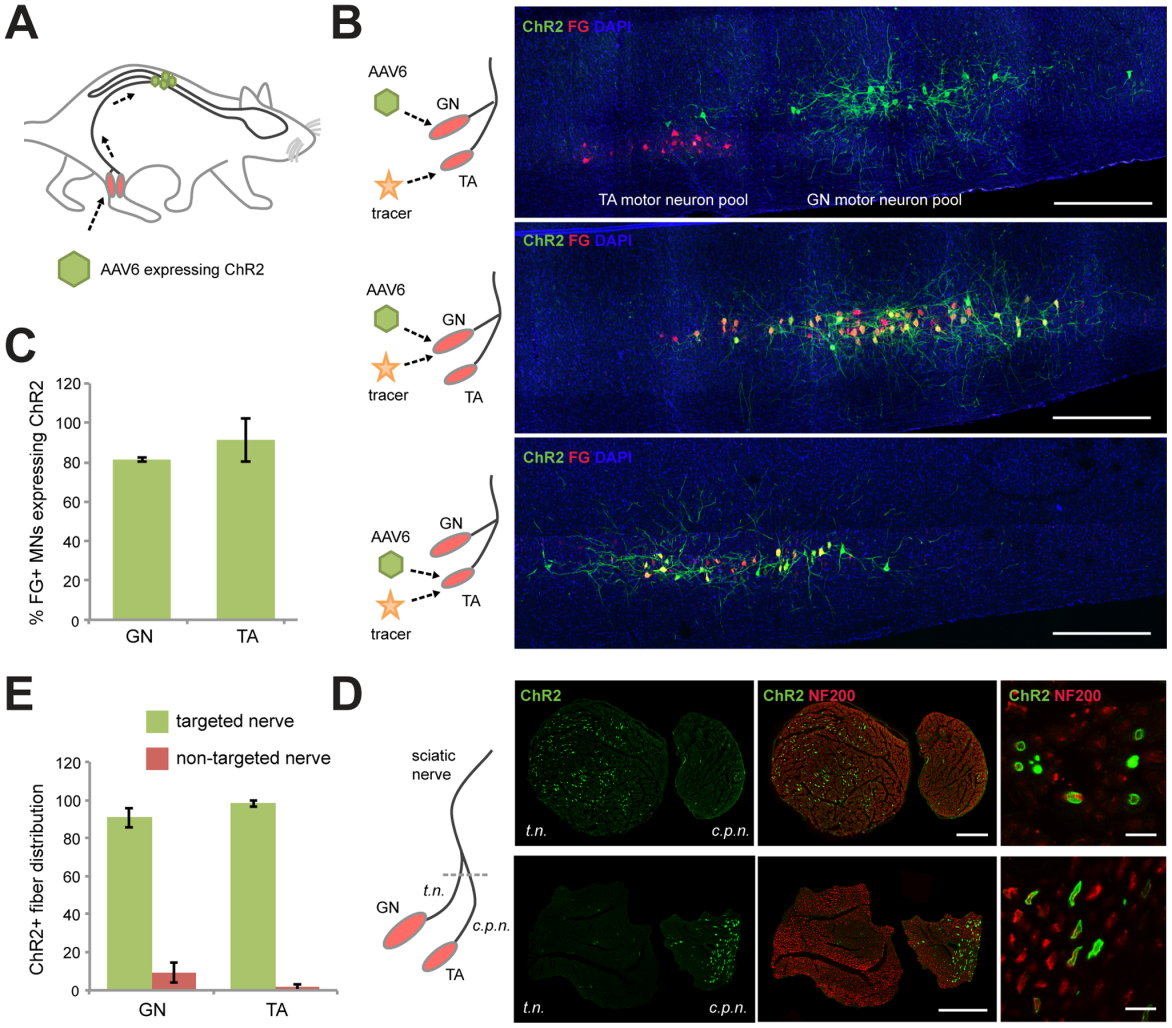
Targeting ChR2 to specific motor neuron axons within the sciatic nerve. (*A*) AAV6 encoding ChR2 fused to yellow fluorescent protein (YFP) was injected into the gastrocnemius muscle (GN) or tibialis anterior (TA) muscle, taken up at the neuromuscular junctions and delivered to spinal cord motor neuron (MN) cell bodies via axonal transport. (*B*) Longitudinal sections of lumbar spinal cord 4 weeks following AAV6 intramuscular injection into GN or TA muscles. The retrograde tracer, Fluoro-Gold (FG), was injected into the ChR2 targeted or non-targeted muscle 1 week prior to analysis. Green, native YFP fluorescence expressed from the ChR2-YFP fusion protein. Red, FG. Blue, DAPI. Scale bar, 1 mm. (*C*) Percentage of FG positive MNs expressing ChR2 in GN (*n* = 5) or TA (*n* = 4). (*D*) Confocal images of sciatic nerve cross-sections following GN or TA muscle injection. The sciatic nerve bifurcates into the tibial nerve (*t.n.*) and common peroneal nerve (*c.p.n.*). Green, ChR2-YFP. Red, Neurofilament 200, a ubiquitous stain for axons. Scale bar, 200 µm. High magnification suggests membrane localization of ChR2. Scale bar, 10 µm. (*E*) Percentage of total ChR2+ axons in the targeted or non-targeted branches of the sciatic nerve following injection in GN (*n* = 5) or TA (*n* = 4).

We injected AAV6 expressing ChR2 (H134R variant) under control of the neuron specific human *synapsin* promoter into either the gastrocnemius (GN) or the tibialis anterior (TA) muscle of Fischer 344 rats, to deliver the ChR2 transgene to the corresponding motor neuron pool in the lumbar spinal cord (Figure 1A) [19]. As the GN and TA muscles are both innervated by the sciatic nerve, this approach allowed us to test if illumination of an entire nerve could specifically activate a sub-population of axons. Three weeks after vector injection, we injected the retrograde tracer, Fluoro-Gold (FG), into the targeted (AAV6-injected) or non-targeted muscle, to quantify motor neuron transduction, and confirm muscle-specific expression of ChR2.

Histological analysis revealed robust ChR2 expression in spinal cord motor neurons one month following AAV6 delivery to the GN or TA muscles (Figure 1B). When FG was injected into the adjacent non-targeted muscle, the FG labeled motor neuron pool and the ChR2-expressing pool were distinct and non-overlapping, demonstrating that retrograde transport of ChR2 was specific to motor neuron pools innervating the injected muscle. When FG was injected into the targeted muscle to quantify motor neuron transduction, the efficiency of retrograde transport was found to be high for both GN motor neurons (81.3±1.2% FG labeled cells expressed ChR2) and TA motor neurons (91.3±11.1% FG labeled cells expressed ChR2) (Figure 1C). We did not observe expression in neighboring interneurons or glial cells suggesting that monosynaptic retrograde transport was the sole mechanism of transduction and that transynaptic spread did not occur. We did, however, observe ChR2 expression in low numbers of large diameter sensory neurons in the dorsal root ganglia (≈1% DRG cells, Figure S1A), possibly as a result of transduction of spindle sensory afferents in the muscle. ChR2 was not expressed in the myofibers of the targeted muscle, as was expected due to use of the neural promoter human *synapsin* (Figure S1B). However, expression was observed in the nerve endings of the neuromuscular junction, demonstrating that the ChR2 was trafficked from cell somata in the spinal cord, along the axons, and to the nerve terminals (a distance of approximately 10 cm) within one month.

We analyzed the sciatic nerve for ChR2 expression. The sciatic nerve bifurcates into the tibial nerve that innervates the GN and the common peroneal nerve that innervates the TA (Figure 1D). ChR2 was strongly expressed in only the nerve branch that projected to the injected muscle. GN injected animals had high numbers of ChR2 expressing axons within the tibial nerve, while TA injected animals favored transduction within the common peroneal nerve (Figure 1E). Greater than ninety percent of opsin-expressing axons were found in the appropriate nerve branch, demonstrating the ability of retrograde transport to deliver ChR2 to targeted sub-populations of axons within a single nerve.

### AAV6:ChR2 delivery enables optogenetic muscle-specific control of the sciatic nerve

**Figure 2:**
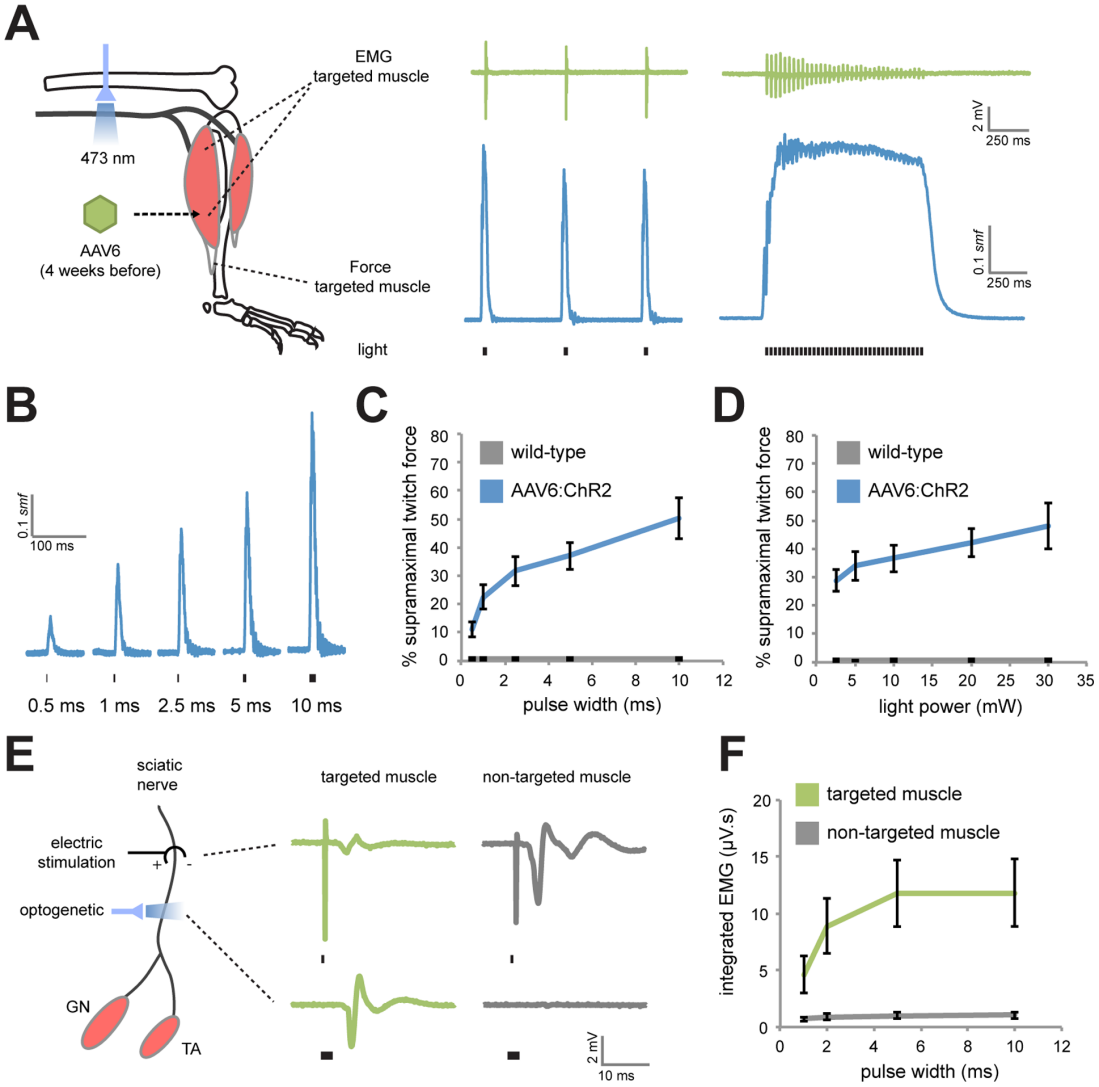
Light-mediated muscle-specific activation of the sciatic nerve. (*A*) Blue light (473 nm) was applied to the sciatic nerve of anesthetized rats 4 weeks following injection of AAV6:ChR2 in the GN or TA. EMG plots show typical responses from optical stimulation taken with fine wire electrodes in the AAV6:ChR2 targeted-muscle (twitch trial: 20 mW, 5 ms, 1 Hz) (tetanus trial: 20 mW, 2.5 ms, 36 Hz). The distal tendon of the muscle was fixed to a transducer to measure force. Representative force traces are shown for corresponding optical activation and are scaled using supramaximal twitch force (*smf*). (*B*) Representative force traces in response to varying pulse widths of 20 mW blue light. (*C*) Percentage of *smf* versus pulse width (20 mW light power) for AAV6:ChR2 (*n* = 7, GN and TA animals combined) or wild-type (*n* = 3) rats. (*D*) Percentage of *smf* versus light power (5 ms pulse width). (*E*) Fine wire electrodes were placed in the targeted and non-targeted muscles of the sciatic nerve. Representative EMG traces are shown following electrical or optogenetic stimulation. (*F*) Integrated EMG versus pulse width (20 mW light power) in the targeted and non-targeted muscles following optical activation (*n* = 9, GN and TA animals combined).

We next examined light-mediated activation of virally expressed ChR2 in motor axons of the sciatic nerve. Four to six weeks after vector delivery, we anesthetized rats and exposed their sciatic nerves. To characterize contractile and electrical responses to optogenetic activation, we attached the distal tendon of the AAV6:ChR2-targeted muscle to a force transducer and placed fine wire electromyographic (EMG) electrodes in both the targeted and non-targeted muscles (Figure 2A). Pulses of blue light (473 nm) applied to the sciatic nerve using an optical fiber were sufficient to evoke robust muscle twitches (Movie S1) and EMG activity in the targeted muscles. High frequency trains of blue light were capable of generating tetanic muscle contractions and corresponding EMG activity. The shape of the force traces for the tetanus trials was dependent on the frequency of the light pulses (Figure S2). Optical activation at greater than 36 Hz resulted in a reduction in force through time and likely reflects a desensitization of ChR2 [20]. We speculate that excitatory opsins with faster kinetics, such as ChETA [21], may be used in the future to overcome this.

The amount of force generated by the AAV6:ChR2-targeted muscle was tunable by modulating light pulse width (range 1 to 10 ms) (Figure 2B, C) or light power (range 2.5 to 30 mW) (Figure 2D). The level of force generated through optogenetic activation exceeded 50% of supramaximal muscle force. We observed no force or EMG activity in response to blue light in wild-type littermates demonstrating that light-responses were not due to the effects of heat on endogenous ion channels.

One advantage of optogenetics over electric stimulation is the ability to control neuronal sub-populations. We measured EMG activity in both the GN and TA muscles during optical and electric stimulation to test whether retrograde transport of AAV6:ChR2 enabled muscle-specific activation of the sciatic nerve (Figure 2E). Indeed, unlike electric stimulation of the sciatic nerve, which resulted in activity in both muscles, optogenetic activation preferentially activated the AAV6:ChR2-targeted muscle across a range of amplitudes (Figure 2F). Optogenetic activation was devoid of the stimulus artifact that accompanies electric stimulation. These results demonstrate targeted optogenetic control of peripheral axon activity in an anesthetized rat.

### Implantable optical nerve cuffs are tolerated and activate motor neurons expressing ChR2

**Figure 3:**
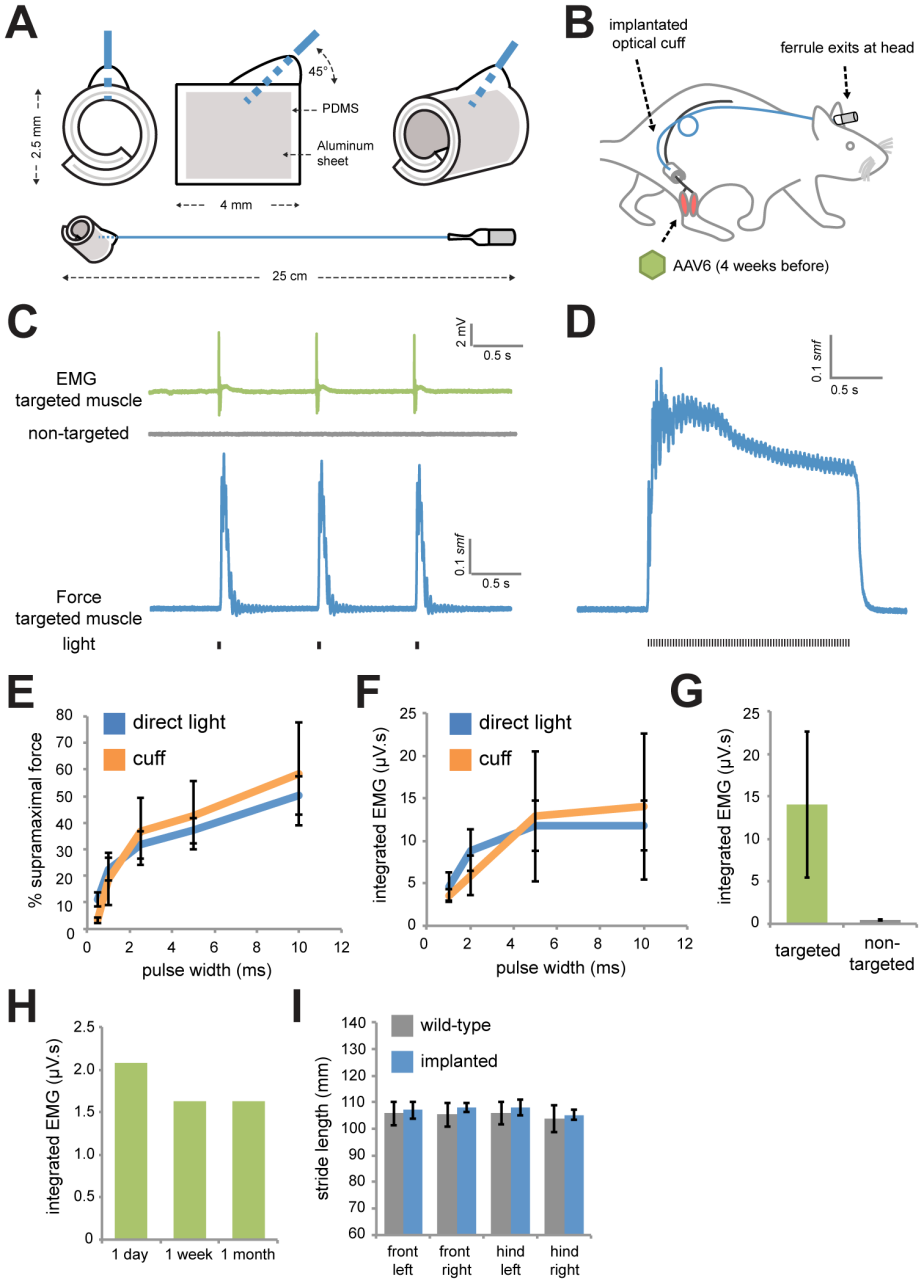
Implantable optical nerve cuffs are well tolerated and activate MNs in anesthetized rats. (*A*) Biocompatible spiral cuffs were constructed from polydimethylsiloxane (PDMS) and covalently bound to a silicon-based optical fiber that was terminated with a stainless steel ferrule. (*B*) Optical nerve cuffs were implanted into rats around the sciatic nerve 4 weeks following AAV6:ChR2 delivery. (*C*) Typical traces of EMG (targeted and non-targeted muscles) and force (targeted muscle only) following illumination using the optical nerve cuff (20 mW, 5 ms, 1 Hz) in anesthetized rats 1 week following cuff implantation. (*D*) Representative force trace following a train of light pulses (20 mW, 2.5 ms, 36 Hz) using the nerve cuff. (*E*) Percentage of *smf* versus pulse width (20 mW light power) using direct laser light application (*n* = 7) or light transmitted through the implanted cuff (*n* = 3). (*F*) Percentage of *smf* versus light power (5 ms pulse width) using direct laser light application (*n* = 7) or light transmitted through the implanted cuff (*n* = 3). (*G*) Integrated EMG in targeted and non-targeted muscles following light delivery using the optical nerve cuff (20 mW, 5 ms) (*n* = 3). (*H*) Integrated EMG in the targeted muscle at 1 day, 1 week, and 1 month following implantation (*n* = 1). (*I*) Stride length of paws in age-matched wild-type littermates (*n* = 4) and rats 1 week post-implantation of optical nerve cuffs (*n* = 5).

We developed an optical fiber-based strategy to direct light from an external light source onto the sciatic nerve of intact rats. We constructed nerve cuffs from two layers of the biocompatible silicon-based organic polymer, polydimethylsiloxane (PDMS) that encased a thin aluminum sheet that served as a reflective surface (Figure 3A). We inserted one end of a silica multimode optical fiber (105 µm core, 125 µm cladding) at a 45° angle into the cuff and covalently coupled it to the cuff using PDMS, terminating the other end using a stainless steel ferrule.

We injected the GN or TA muscles of rats with AAV6:ChR2 and once ChR2 expression was established, three to five weeks later, implanted these rats with the optical nerve cuff (Figure 3B). We wrapped the cuff around the sciatic nerve and tunneled the optical fiber under the skin up to the head, cementing the ferrule to the skull. One week after implantation, we measured muscle force and EMG signals of the rats under anesthesia to determine if light delivery through the optical nerve cuff was possible following a week of free animal movement. We observed consistent light-mediated generation of twitch (Figure 3C) and tetanic force (Figure 3D) through the optical nerve cuff. Force and EMG activity was similar to that achieved with direct light application and was tunable by modulating light pulse width (Figure 3E) and light power (Figure 3F). EMG recordings demonstrated that the optical nerve cuff preferentially activated the targeted muscle over the non-targeted muscle (Figure 3G). We also observed that the optical nerve cuff could elicit muscle activation for at least 4 weeks following implantation (Figure 3H).

To test whether the optical nerve cuff was tolerated by rats, we performed automated gait analysis of the rats at one week post-implantation. Gait is sensitive to motor and sensory neuron damage, with gait changes arising in response to nerve crush [22], nerve transection [23] and nerve constriction [24]. The nerve cuffs did not alter stride length (Figure 3I) or the directedness of steps (coupling symmetry) (Figure S3), suggesting that the cuff did not interfere with normal movement or nerve function. In addition, we observed no overt signs of discomfort over the month, and the rats exhibited normal behavior.

### Optical nerve cuffs modulate motor neuron activity in freely moving animals

**Figure 4:**
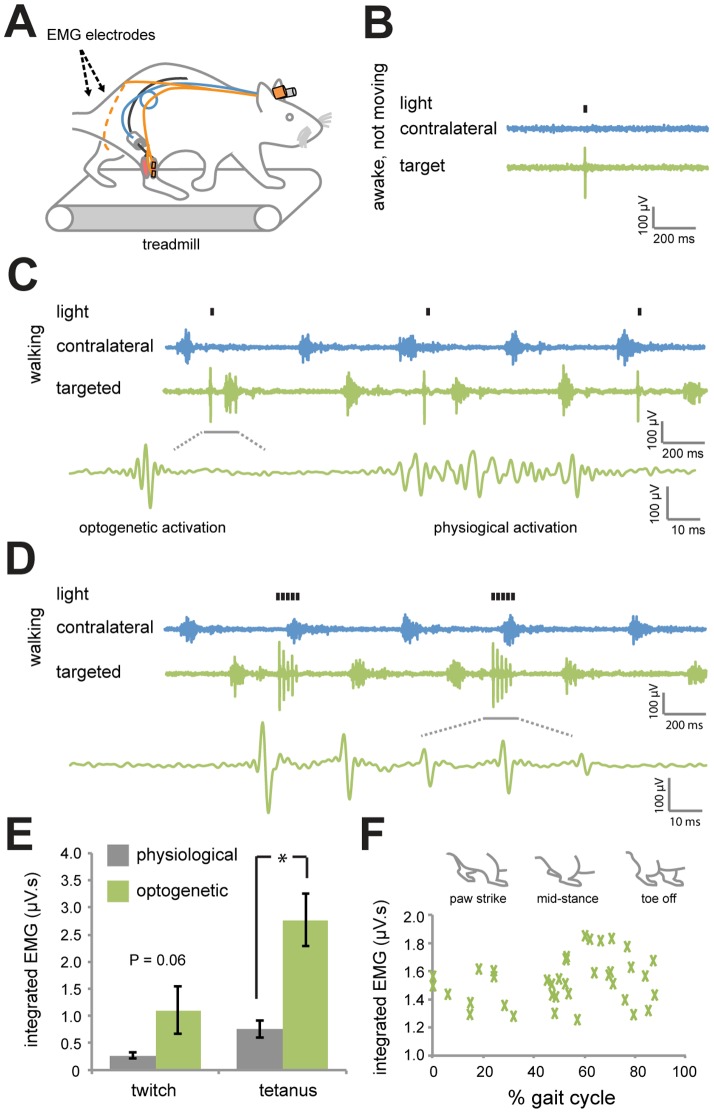
Optogenetic activation of targeted sciatic nerve axons in awake and moving rats. (*A*) Optical nerve cuffs and EMG electrodes were implanted into rats 4 weeks following AAV6:ChR2 delivery. EMG electrodes were implanted onto the surface of the AAV6:ChR2-injected muscle (targeted) and onto the uninjected muscle on the opposite leg (contralateral). Non-anesthetized rats were tested for EMG activity on a treadmill 3 days following the surgery. (*B*) EMG activity in response to a pulse of blue light (20 mW, 5 ms) in the targeted and contralateral muscles in awake, non-moving rats. (*C*) EMG activity in response to pulses of light (20 mW, 5 ms, 1 Hz) in awake rats walking on a treadmill at constant speed (20 cm/s). (*D*) EMG activity in response to 150 ms trains of light (20 mW, 5 ms, 36 Hz) in awake rats walking on a treadmill. (*E*) Integrated EMG in the targeted muscle in response to optogenetic or physiological activation (*n* = 3, animals matched) for twitch and tetanus contractions. Integrated EMG responses following optogenetic activation are greater or equal to physiological activity (* *P*<0.05; 2 tailed paired T-test). (*F*) Integrated EMG versus gait cycle demonstrating that activity was independent of the position of the legs (*n* = 32 trials, *R^2^* = 0.05).

We assessed whether the optical nerve cuffs could modulate PNS activity in freely moving rats using implanted EMG electrodes to record muscle activity. We sutured electrodes to the surface of the AAV6:ChR2-injected muscle and non-injected contralateral muscle (immediately following cuff implantation) and tunneled wires subcutaneously to a pedestal mounted at the skull (Figure 4A). We observed consistent muscle twitches (Movie S2) and EMG activity in response to blue light (20 mW light power, 5 ms pulse width) in non-moving, but awake rats (Figure 4B). In wild-type animals implanted with the optical nerve cuff and electrodes, muscles were not activated by illumination of the sciatic nerve with blue light.

We then applied optogenetic activation while the rats were walking on a motorized treadmill to test whether optogenetic control could be achieved in freely moving animals (Movie S3). Illumination with blue light (20 mW light power, 5 ms pulse width, 1 Hz pulse frequency) resulted in EMG spikes that were distinguishable from physiological activity (Figure 4C). These spikes were not observed in the contralateral muscle. We then applied trains of light pulses to simulate tetanic contraction used by the targeted muscle during ambulation. We used 36 Hz optogenetic trains (20 mW light power, 5 ms pulse width) to generate tetanus (Figure S2) and applied this stimulus over 150 ms, the average time of physiological muscle activation in the targeted muscles during ambulation. 150 ms of EMG activity could be achieved (Figure 4D), demonstrating temporally relevant optogenetic muscle control in freely moving animal.

To compare optogenetic and physiological activation, we calculated integrated EMG (iEMG) values for the various trials. We observed that light-mediated activation (20 mW light power, 5 ms pulse width) generated equal or larger iEMG than physiological activity over equivalent periods (Figure 4E) (*P*<0.05), although there were important differences in synchronicity, which could confound a direct comparison of iEMG. Furthermore, iEMG values for individual twitches were independent of gait cycle position (Figure 4F), suggesting that light delivery by the cuff to the nerve was not significantly affected by rat leg position. These findings demonstrate that optogenetic activation using the optical nerve cuff can consistently achieve muscle activation in freely moving animals.

## Discussion

We have developed an approach that enables optogenetic modulation of the PNS. We have shown that retrograde transport of muscle-injected AAV6:ChR2 results in expression of ChR2 in a targeted motor neuron pool. Illumination of peripheral nerves containing these targeted motor axons in rats results in muscle activation that is restricted to the targeted muscle, unlike nonspecific electrical stimulation with a single electrode. We have also shown that chronically implanted optical nerve cuffs can be used to control motor neuron activity, enabling optogenetic modulation of peripheral axon activity in awake and behaving animals.

The optogenetic approach presented here has several advantages over conventional muscle activation with electrical nerve cuffs. For example, physical constraints can limit the degree of selectivity of muscle activation with electrical nerve cuffs. Many muscles, such as the vastus intermedius or piriformis muscles, are innervated by nerve branches that are too short, too deep or too highly branched for placement of a cuff at the innervation site. The viral-based approach, however, would allow the stimulation cuff to be placed in a convenient location (in a mixed nerve area) while retaining selectivity for the targeted muscle. In addition, activation of motor neurons can be achieved in a mixed nerve with minimal sensory neuron recruitment (Figure S1A). Controlling muscles independently would allow researchers to probe the contribution of specific muscles to gait during normal function or after a neurological injury such as stroke. Furthermore, chronic optogenetic stimulation of specific muscles without any confounding non-specific activation may provide insight into how surrounding non-stimulated muscles compensate for excess activity in a nearby muscle.

The present study achieves optogenetic control of peripheral nerves through use of recombinant viral vectors, unlike previous reports that required the use of transgenic animals [3]–[7]. Vector gene delivery has several advantages over transgenesis. Perhaps most importantly, viral vectors can be readily adapted to new opsins, unlike transgenic animals, which need to be created for each separate opsin. Given the rapid expansion of the optogenetic toolbox [25], with resultant continuous refinement of opsin sensitivity and spectral profile, it is critical that any optogenetic technique be able to quickly take advantage of such innovation. Secondly, viral opsin delivery can be combined with currently existing transgenic models of peripheral nervous disease, without the need for careful breeding, thereby expanding the scope of optogenetic modulation to the study of many disorders of the peripheral nervous system. Finally, while transgenesis is not readily adaptable to larger species, viral vectors have been successfully used in non-human primates and in humans [19], [26]. The eventual translation of optogenetic techniques to the primate peripheral nervous system therefore requires the development of viral opsin delivery strategies such as those presented in this paper.

The methods described here can be applied to more complex interventions. We have demonstrated that retrograde transport of AAV6 confers muscle specificity. In principle therefore, if two spectrally separated opsins (such as ChR2 and the red-shifted chimera C1V1 [27], [28]) were injected into two antagonistic muscles, a single optical cuff could be used to bi-directionally control a joint. In larger animals, multiple nerve cuffs could be implanted around nerve branches and complex multi-wavelength optical stimulation used to artificially re-animate entire limbs. Such optogenetic modulation need not be restricted to activation. An AAV6 expressing an inhibitory opsin, such as halorhodopsin (NpHR) [29] or archaerhodopsin (Arch) [30], could be used instead of ChR2 to examine if optogenetic inhibition of motor neuron axons in animal models of spasticity is sufficient to eliminate or reduce spastic hypertonia.

Optogenetics can be applied to study other peripheral processes beyond modulation of motor neurons. Various serotypes of AAV and herpes simplex virus are commonly used to deliver transgenes to sensory neurons following retrograde transport [14], [16]. These viruses could be used to transduce nociceptive neurons with excitatory or inhibitory opsins, allowing for study of the effects of modulation of these infected nerves on the development and treatment of a variety of somatosensory and pain-related disorders. While the current study used the pan-neuronal human *synapsin* promoter, where available, other promoters could be substituted to achieve additional intersectional specificity in cases where retrograde transport from a peripheral tissue is not sufficient to confer selective targeting of the desired neural type. Finally, the use of an optical nerve cuff as opposed to transdermal light delivery [6] allows further specificity defined by nerve anatomy and modulation of deeper neuronal structures such as autonomic nerves and ganglia, thereby enabling the translation of peripheral optogenetic techniques to larger animals such as primates.

## Materials and Methods

### Viral vector

AAV6 expressing ChR2 (AAV6:ChR2) was produced at the University of North Carolina Vector Core Facility. The expression cassette comprised ChR2 (H134R mutant) fused to yellow fluorescent protein (YFP) under control of the human *synapsin* promoter. Genomic DNA was packaged into the AAV6 capsid using helper plasmids. One viral batch was used with a dot-blot hybridization titer of 3.2×10^13^ vg/mL.

### Ethics Statement

Animal procedures were approved by the Stanford Institutional Animal Care and Use Committee.

### Animals and intramuscular injection

Fischer 344 rats (Charles River Laboratories, Wilmington, MA) were housed under a 12∶12 light∶dark cycle in a temperature-controlled environment with food and water available *ad libitum*. Intramuscular injections were made in adult and newborn rats. Tibialis anterior (TA) injections were performed on 8-week-old female rats. Animals were anesthetized using isoflurane and an incision made to expose the TA. Twenty-five microliters of AAV6:ChR2 (6.4×10^11^ vg) were delivered across three sites using a 30 G beveled needle at 5 µL/min. The skin was closed with suture. Gastrocnemius (GN) injections were performed on neonate rats one or two days postpartum as this mode of gene delivery results in high levels of expression and may facilitate optogenetic studies during development. Pups were anesthetized with hypothermia and each head of the GN injected with 0.75 µL (1.5 µL total) of AAV6:ChR2 (1.25×10^10^ vg) using a 35 G beveled needle (World Precision Instruments, Sarasota, FL) connected to a syringe (Hamilton, Reno, NV) with polyethylene tubing (A–M Systems, Carlsborg, WA). One week prior to sacrifice, animals were injected with Fluoro-Gold (FG, Fluorochrome, Denver, CO) in the AAV6-injected muscle or the adjacent non-AAV-injected muscle. Twenty-five microliters of 4% FG in phosphate buffered saline (PBS) was delivered across three sites of either the GN or TA using a 30 G beveled needle at 5 µL/min. The skin was closed with suture.

### *In vivo* animal preparation and acute stimulation and recording

Animals expressing ChR2 were anesthetized using isoflurane and warmed with a heating pad. The hindlimb and back of the animal was shaved. The bone attached to the proximal tendon of the targeted muscle was immobilized. The muscle was exposed and kept moist with Ringer's solution. The distal tendon of the muscle was cut and tied to a force transducer (Aurora Scientific, Aurora, Ontario, Canada, 300C-LR) with nylon suture. A differential electrode arrangement was used to measure electromyographical activity (EMG) of the targeted muscle and the opposing non-targeted muscle. If the animal was not previously implanted with EMG electrodes, fine wire electrodes (A.M. Systems, Cat: 790900, Sequim, WA) were inserted into the muscle belly and distal to the muscle belly. A common ground wire was inserted into the base of the tail of the rat. If the animal was not previously implanted with an optical nerve cuff, the sciatic nerve was exposed by blunt dissection of the thigh muscles. An optical fiber was positioned perpendicular to the axis of the nerve such that the diameter of the light spot was equivalent to the diameter of the nerve (1 mm). For electrical stimulation, two wires were wrapped around the nerve. The sciatic nerve was kept moist with Ringer's solution. After *in vivo* experiments, animals were sacrificed for histology.

Electrical stimulation (Grass Technologies, West Warwick, RI, S48) was used to determine supramaximal force, defined as the maximum twitch force achievable with 0.1 ms electrical pulse of the sciatic nerve. Responses to optical stimulation (East Lansing, MI, MBL-III473), both twitch and tetanic, were measured at various light pulse widths (0.5–10 ms) and powers (1–30 mW). Tetanic stimulation was performed with a range of frequencies (24–100 Hz). Muscle tension and EMG were recorded at 10 kHz for the duration of stimulation (National Instruments, Austin, TX, PCI-6251).

### Optical nerve cuff construction

Polydimethylsiloxane (PDMS) (Dow Corning, Midland, MI, Sylgard® 184 Silicone Elastomer) was mixed with a curing agent to base polymer ratio of 1∶30 and bubbles removed under vacuum. The PDMS was poured to 0.4 mm thickness and cured. The cured 1∶30 PDMS was stretched uni-directionally to twice its original length. A 2.5 mm×5 mm rectangle of aluminum sheet (0.016 mm thickness) was embedded between the stretched layer of PDMS and 0.1 mm thick layer of uncured PDMS (1∶10 ratio of curing agent to base polymer). After curing, the stretched layer was released, and the assembly coiled into a spiral cuff shape, similar to previously described electrical stimulating implants [18]. The cylindrical cuff was cut to size, 4 mm length with edges overlapping 2 mm. An additional hemisphere of PDMS was added to the cuff, opposite the overlapping edges as shown in Figure 3A. A 25 cm optical fiber (Thor Labs, Newton, NJ, AFS105/125Y) was terminated with a stainless steel ferrule (Precision Fiber Products, Milpitas, CA, MM-FER2003SS-1260) and polished. A 1 cm length of polyolefin heat shrink was used to relieve strain between the ferrule and the fiber. The distal end of the fiber was terminated with a clean break, and the jacket of the fiber was stripped 1.5 mm. The distal end of the fiber, coated in uncured PDMS, was inserted through the additional hemisphere on the cuff at a 45° angle and the PDMS was cured. The optical nerve cuffs were then sterilized by autoclave.

### Implantation of cuffs and EMG electrodes

Animals were anesthetized with isoflurane, placed in the prone position, and warmed using a heating pad. An injection of buprenorphine was given for analgesia. The head, back, and leg of the animal were shaved and the skin disinfected using povidone-iodine and isopropyl alcohol. Sterile surgical procedures were followed. A midline incision was made to expose the skull. The skull was scraped with a scalpel blade and allowed to dry before a layer of Metabond (Parkell, Edgewood, NY) was applied. The sciatic nerve was exposed by skin incision and blunt separation of the thigh muscles. A plastic tube (5 mm outer diameter, 3 mm inner diameter) was tunneled subcutaneously from this incision to the head incision. The nerve cuff implant was inserted ferrule end first into the caudal end of the tube and transferred rostrally. The tube was removed leaving the implant in the animal. The optical fiber was looped once to provide strain relief (Figure 3B). The optical nerve cuff was placed around the sciatic nerve such that it did not constrict or deform the nerve. The optical fiber was secured at a position that minimized nerve strain with two loose sutures. The sciatic nerve pocket was closed using absorbable suture. The ferrule was cemented to the head with Metabond at a horizontal angle of approximately 30° and stabilized using generic dental cement.

In some animals, implantable stainless steel electrodes (Plastics One, Cat: E363/76H, Roanoke, VA) were implanted directly after implantation of the cuff. The electrodes were sutured to the surface of the target muscle and the contralateral muscle on the opposite leg. A ground electrode was sutured to the base of the tail. The proximal ends of these electrodes were inserted into a plastic pedestal (Plastics One, Cat: MS363, Roanoke, VA), which was cemented to the skull in the same fashion as the ferrule. The skin was sutured on the head and legs and final injections of antibiotics (enrofloxacin), anti-inflammatories (carprofen) and local anesthetics (bupivacaine) were given.

### Stimulation in awake and moving rats

Rats were trained to walk on a treadmill (Harvard Apparatus, Holliston, MA, 760303) at 25 cm/s for 10 minutes per day, 2 days per week for 2 weeks before implantation of the nerve cuff. At time of testing, a fiber optic cable and ceramic sleeve were used to connect the laser to the nerve cuff ferrule at the skull. Custom cabling (Plastics One, Cat: 363–491/6, Roanoke, VA) connected the data acquisition setup to the EMG electrode pedestal. EMG activity was first monitored while the animal was awake, but not moving, both with and without light stimulation. EMG activity was then gathered while the animal walked at 25 cm/s, both with and without stimulation. Light stimulation included single pulses as well as trains of light. A 2-tailed Student's T-test was used to compare the means of the physiological or optogenetic integrated EMG for twitch and tetanus trials.

### EMG processing and acquisition

EMG signals were filtered (0.01–30 Hz) and amplified (50×) in hardware (Grass Technologies, P511 AC Amplifier) and acquired through a DAQ (National Instruments, BNC-2090A, Austin TX) controlled through custom scripts written in MATLAB (The MathWorks, Natick, MA). When required, signals were filtered in MATLAB (5–500 Hz). For quantification, EMG segments immediately following optical stimulation were isolated, rectified, and integrated to obtain iEMG values.

### Gait testing for tolerability

Gait was examined one week following implantation using the Catwalk XT gait analysis system (Noldus Information Technology, Asheville, NC). Rats ambulated on an illuminated glass surface within a narrow corridor. Footprints were recorded with a high-speed camera. Five trials were acquired per animal and analyzed using the Catwalk XT software. Stride length and coupling symmetry were compared with non-implanted age-matched littermates. Means were analyzed for statistical differences using Student's T-test.

### Histology

Rats were anesthetized and perfused with 4% paraformaldehyde (PFA) in PBS. Spinal cord, muscle, sciatic nerve, and dorsal root ganglia were dissected, postfixed (4°C overnight), and transferred to 30% sucrose in PBS (4°C until sectioning). Tissues were embedded in Tissue-Tek OCT compound (Sakura, Holland) and sectioned on a Leica CM3050 cryostat (Leica, Buffalo Grove, IL). Spinal cords and muscle were cut in longitudinal 40 µm sections and mounted onto glass slides. Slides were stained using DAPI and mounted in PVA-DABCO (Sigma-Aldrich, St Louis, MO). Sciatic nerve and ganglia were processed in the same manner with 20 µm section thickness. Nerves were stained using mouse antibody against neurofilament (NF200, 1∶200, Sigma) and interrogated with a Cy3-congugated goat anti-mouse IgG Ab (1∶1000, Jackson ImmunoResearch Laboratories, West Grove, PA) prior to mounting. Images were taken with an SP5 Confocal Microscope (Leica). The percentage of FG positive motor neurons expressing ChR2 was quantified in three out of four sections. The number of ChR2 positive axons in the targeted branch of the sciatic nerve (i.e. *c.p.n.* for TA injections or *t.n*. for GN injections) was quantified and expressed as a percentage of total ChR2 positive axons in both branches of the sciatic nerve.

## Supporting Information

Figure S1 Opsin expression in dorsal root ganglia and muscle. (*A*) Confocal images of L5 dorsal root ganglia (DRG) 4 weeks following AAV6:ChR2 intramuscular injection into GN or TA muscles. Green, native YFP fluorescence expressed from the ChR2-YFP fusion protein. Blue, DAPI. Scale bar, 200 µm. (*B*) Confocal images of muscle from targeted (AAV6-injected) or non-targeted muscles following delivery into GN or TA muscles. Scale bar, 200 µm. No expression was observed in muscle fibers, however, high magnification reveals expression of ChR2 in nerve endings within the muscle. Scale bar, 40 µm.
(TIF)

Figure S2 The effect of optical train frequency on ChR2 muscle activation. Typical EMG and force traces following pulses of blue light (20 mW, 2.5 ms) at 24, 36, 50, and 100 Hz.
(TIF)

Figure S3 The effect of implanted nerve cuffs on gait. Coupling symmetry of paws in age-matched wild-type littermates (*n* = 4) and rats 1 week post-implantation of optical nerve cuffs (*n* = 5). HL, hind left, FL, front left, HR, hind right, FR, front right. There is no significant difference between wild-type and implanted animals. *P* values, HL−HR = 0.30, FL−FR = 0.97, FL−HL = 0.19, HR−FR = 0.26.
(TIF)

Movie S1 Optogenetic activation of targeted muscles in an anesthetized rat. AAV6:ChR2 was injected into the gastrocnemius five weeks earlier. The gastrocnemius was attached to a force transducer through the Achilles' tendon and had EMG wires placed in the belly of the muscle. Six pulses of blue light (20 mW light power, 5 ms pulse duration, 1 Hz) were directed onto the sciatic nerve resulting in contraction in the gastrocnemius muscle.
(MOV)

Movie S2 Optogenetic activation of targeted muscles in an awake rat. AAV6:ChR2 was injected into the tibialis anterior, and four weeks later an optical nerve cuff was implanted around the sciatic nerve. Light was delivered to the cuff three days following implantation (20 mW light power, 10 ms pulse duration, 1 Hz) in the awake animal in multiple resting positions. Note that illumination caused dorsiflexion of the paw which is expected for activation of the tibialis anterior.
(MOV)

Movie S3 Experimental setup for treadmill experiment. AAV6:ChR2 was injected into the tibialis anterior or gastrocnemius, and four to six weeks later, an optical nerve cuff was implanted around the sciatic nerve. Surface EMG electrodes were also implanted on the targeted muscle and the non-injected contralateral muscle on the opposite leg. Rats were trained to use the treadmill, and 3 days following cuff/EMG implantation, light was applied during walking on the treadmill. Muscle twitches are not observable in the video (due to gross limb movements, see instead Movie S2), however, the EMG data demonstrates distinct optogenetic activation of the targeted muscle (Figure 4B).
(MOV)
