# Supplementary figures and images for: Optogenetic Control of Targeted Peripheral Axons in Freely Moving Animals
Source: PLoS One. 2013 Aug 21;8(8):e72691. doi: 10.1371/journal.pone.0072691 (PMC3749160; doi:10.1371/journal.pone.0072691)

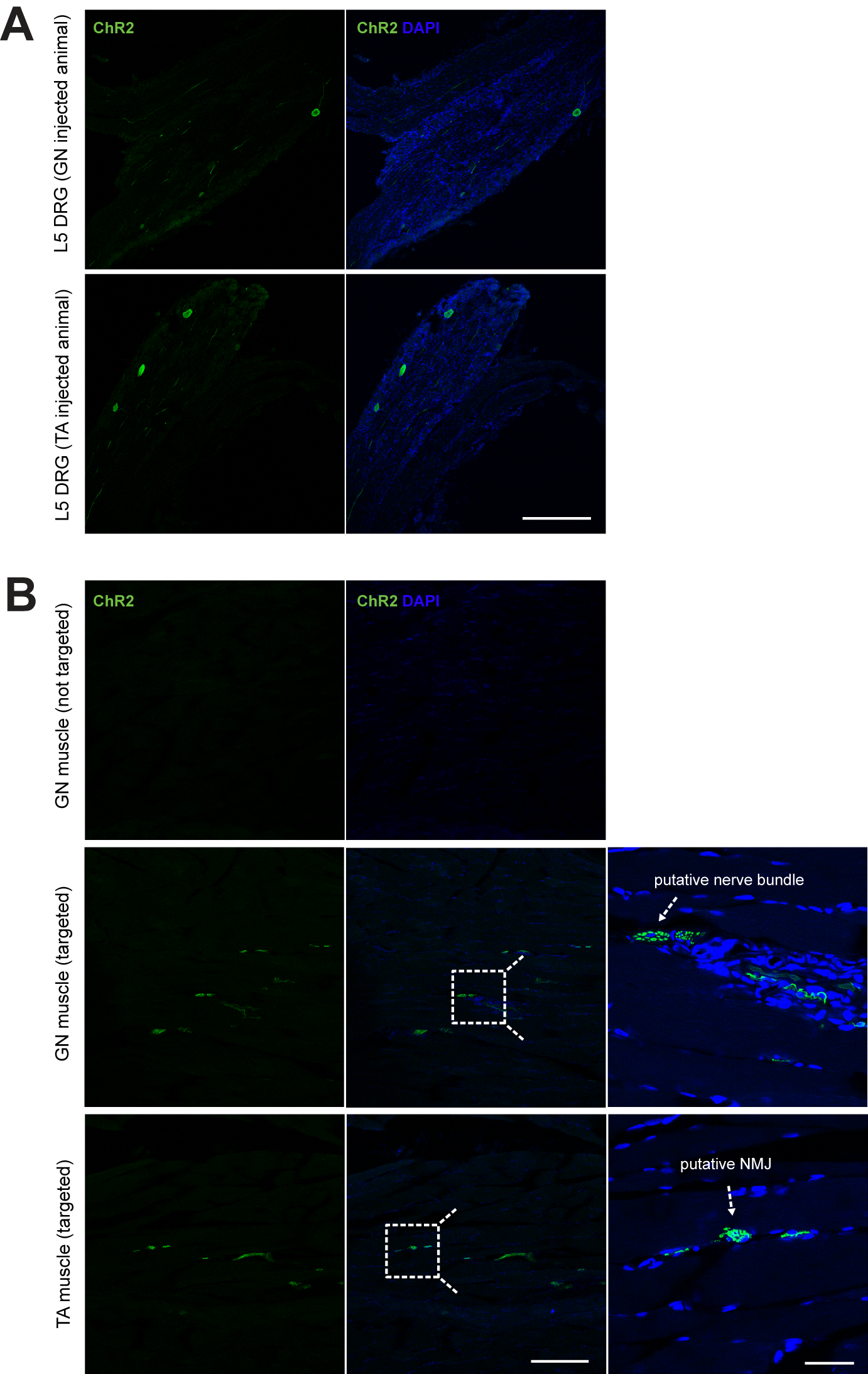

Supplement: Figure S1 — Opsin expression in dorsal root ganglia and muscle. (A) Confocal images of L5 dorsal root ganglia (DRG) 4 weeks following AAV6:ChR2 intramuscular injection into GN or TA muscles. Green, native YFP fluorescence expressed from the ChR2-YFP fusion protein. Blue, DAPI. Scale bar, 200 µm. (B) Confocal images of muscle from targeted (AAV6-injected) or non-targeted muscles following delivery into GN or TA muscles. Scale bar, 200 µm. No expression was observed in muscle fibers, however, high magnification reveals expression of ChR2 in nerve endings within the muscle. Scale bar, 40 µm. (TIF) [file pone.0072691.s001.tif]

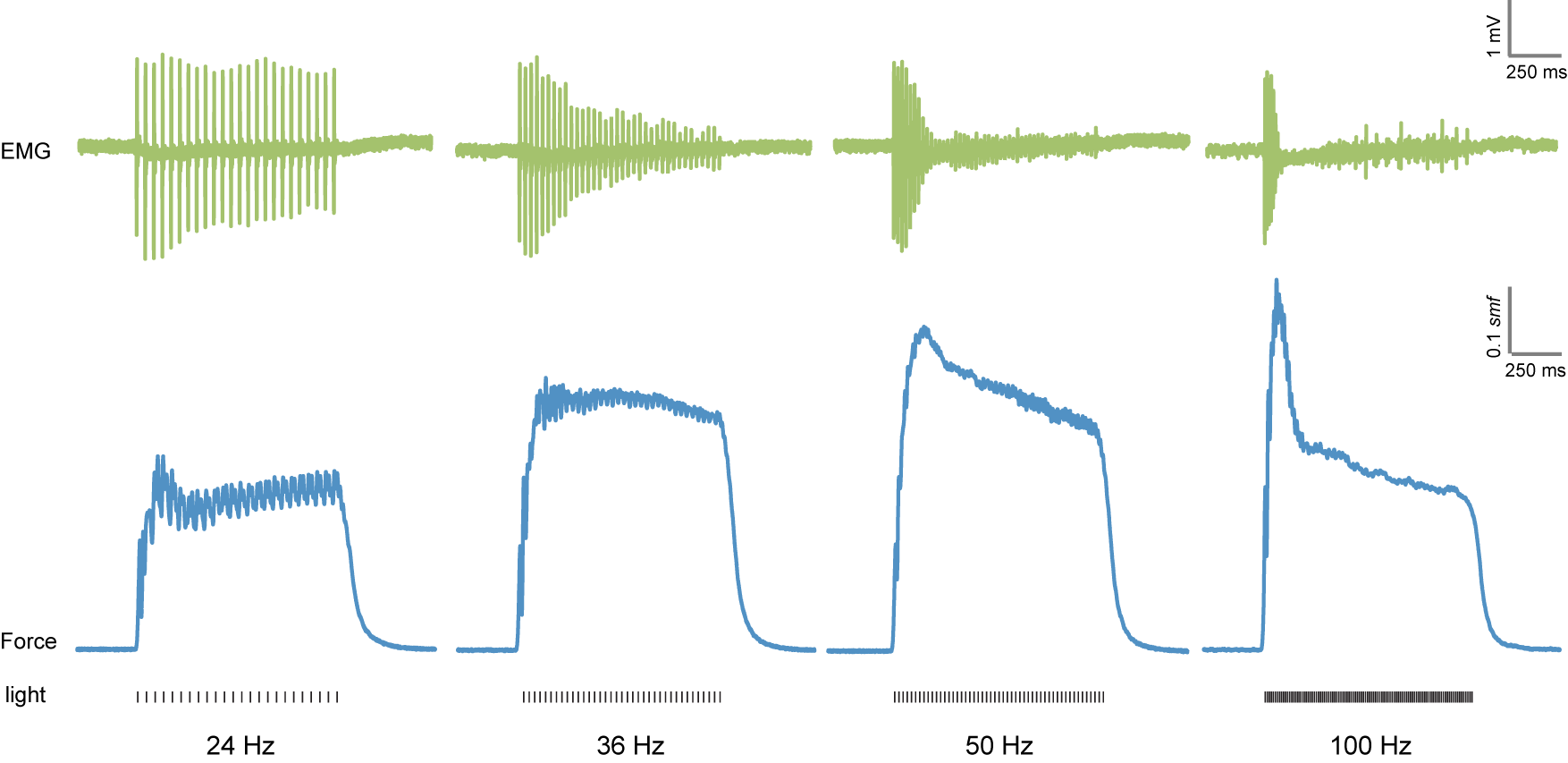

Supplement: Figure S2 — The effect of optical train frequency on ChR2 muscle activation. Typical EMG and force traces following pulses of blue light (20 mW, 2.5 ms) at 24, 36, 50, and 100 Hz. (TIF) [file pone.0072691.s002.tif]

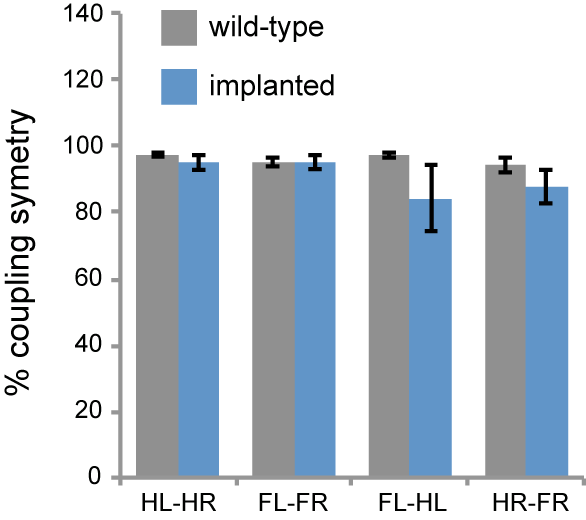

Supplement: Figure S3 — The effect of implanted nerve cuffs on gait. Coupling symmetry of paws in age-matched wild-type littermates (n = 4) and rats 1 week post-implantation of optical nerve cuffs (n = 5). HL, hind left, FL, front left, HR, hind right, FR, front right. There is no significant difference between wild-type and implanted animals. P values, HL−HR = 0.30, FL−FR = 0.97, FL−HL = 0.19, HR−FR = 0.26. (TIF) [file pone.0072691.s003.tif]
